# Management and Molecular Characterization of Intraventricular Glioblastoma: A Single-Institution Case Series

**DOI:** 10.3390/ijms241713285

**Published:** 2023-08-27

**Authors:** Megan Parker, Anita Kalluri, Joshua Materi, Sachin K. Gujar, Karisa Schreck, Debraj Mukherjee, Jon Weingart, Henry Brem, Kristin J. Redmond, Calixto-Hope G. Lucas, Chetan Bettegowda, Jordina Rincon-Torroella

**Affiliations:** 1 Department of Neurosurgery, Johns Hopkins University School of Medicine, Baltimore, MD 21205, USA; 2 Division of Neuroradiology, The Russell H. Morgan Department of Radiology and Radiological Science, Johns Hopkins University School of Medicine, Baltimore, MD 21205, USA; 3 Department of Neurology, Sidney Kimmel Comprehensive Cancer Center, Johns Hopkins University School of Medicine, Baltimore, MD 21287, USA; 4 Department of Radiation Oncology and Molecular Radiation Sciences, Johns Hopkins University School of Medicine, Baltimore, MD 21205, USA; 5 Department of Pathology, Johns Hopkins University School of Medicine, Baltimore, MD 21205, USA

**Keywords:** glioblastoma, intraventricular, next-generation sequencing, IDH-wildtype

## Abstract

While the central nervous system (CNS) tumor classification has increasingly incorporated molecular parameters, there is a paucity of literature reporting molecular alterations found in intraventricular glioblastoma (IVGBM), which are rare. We present a case series of nine IVGBMs, including molecular alterations found in standardized next-generation sequencing (NGS). We queried the clinical charts, operative notes, pathology reports, and radiographic images of nine patients with histologically confirmed IVGBM treated at our institution (1995–2021). Routine NGS was performed on resected tumor tissue of two patients. In this retrospective case series of nine patients (22% female, median (range) age: 64.3 (36–85) years), the most common tumor locations were the atrium of the right lateral ventricle (33%) and the septum pellucidum (33%). Five patients had preoperative hydrocephalus, which was managed with intraoperative external ventricular drains in three patients and ventriculoperitoneal shunts in one patient. Hydrocephalus was managed with subtotal resection of a fourth ventricular IVGBM in one patient. The most common surgical approach was transcortical intraventricular (56%). Gross total resection was achieved in two patients, subtotal resection was achieved in six patients, and one patient received a biopsy only. Immunohistochemistry for IDH1 R132H mutant protein was performed in four cases and was negative in all four. Genetic alterations common in glioblastoma, IDH-wildtype, were seen in two cases with available NGS data, including EGFR gene amplification, TERT promoter mutation, PTEN mutation, trisomy of chromosome 7, and monosomy of chromosome 10. Following surgical resection, four patients received adjuvant chemoradiation. Median survival among our cohort was 4.7 months (IQR: 0.9–5.8 months). Management of IVGBM is particularly challenging due to their anatomical location, presentation with obstructive hydrocephalus, and fast growth, necessitating prompt intervention. Additional studies are needed to better understand the genetic landscape of IVGBM compared to parenchymal glioblastoma and may further elucidate the unique pathophysiology of these rare tumors.

## 1. Introduction

Glioblastoma (GBM) is the most common and aggressive adult CNS malignancy. The median age at presentation is 64 years, and the median survival is 15 months [1,2]. GBMs are typically located in the parenchyma of the cerebral hemispheres but can very rarely present in the ventricular system [3,4,5,6,7,8,9,10,11,12,13,14,15,16,17]. There are several primary brain tumors including central neurocytoma, ependymoma, subependymoma, and choroid plexus tumors that frequently arise within the ventricles [18]. However, GBM arising within the ventricular system is exceedingly rare. Most recently, Nsir et al. reported less than 30 cases of (IVGBM) in their literature review and case series [19]. Additionally, while CNS tumor diagnosis and classification has increasingly incorporated molecular parameters, there is limited information about the genetic alterations seen in IVGBM [20]. Here, we present a case series of nine IVGBMs, including the molecular characterization of a subset of cases.

## 2. Materials and Methods

We retrospectively analyzed the clinical charts, operative notes, pathology reports, and radiographic images of 9 consecutive patients diagnosed with histologically confirmed IVGBM who were treated at our institution between 1995 and 2021. This study was approved by the Johns Hopkins Hospital institutional review board (IRB00308655).

Patients were identified via institutional surgical pathology records and neurosurgical databases from March 1984–August 2021. The search criteria were “glioblastoma”, “intraventricular”, and “neurosurgical intervention”. Intraventricular was defined as a tumor that arose from the intraventricular lining/walls or adjacent brain parenchyma that extended into the ventricular system and lay primarily within the ventricular system based on surgical and radiographic reports. All cases were reviewed by expert neuroradiologists and neurosurgeons. Formalin-fixed, paraffin-embedded (FFPE) tumor tissue specimens were reviewed by expert neuropathologists at our institution. Intraventricular tumors that were initially given a histopathological diagnosis of “glioblastoma” were included in the study. H&E stained slides for 7 patients were re-reviewed and assigned a grade according to the WHO 2016 classification of central nervous system tumors by an expert neuropathologist [21]. IDH mutation status was not available for all patients; therefore, we could not assign a grade according to the WHO 2021 classification of central nervous system tumors [20]. FFPE blocks were not available for patients 5 and 9 for re-review, so information from the original surgical pathology reports was used to classify these tumors. GBM was defined by the presence of atypical glial cells with an increased mitotic rate (>5 mitotic figures per 10 high-power fields). Tumors with microvascular proliferation and/or necrosis were assigned a grade IV designation, according to the WHO 2016 classification of central nervous system tumors [21].

In two cases, genomic DNA was extracted from macro-dissected FFPE tumor tissue. Molecular tests performed included MGMT promoter methylation, trisomy of chromosome 7, monosomy of chromosome 10, and next-generation sequencing (NGS). Molecular analysis of the MGMT gene was performed by methylation-specific PCR. The MGMT and beta-Actin copy numbers were used to calculate the ratio of MGMT/beta-Actin × 1000 (Unmethylated < 2.00, Methylated ≥ 2.00). NGS was performed on resected tumor tissue at the Johns Hopkins Medical Laboratories [22]. The incorporation of routine NGS of clinical samples into the diagnostic workup occurred around 2017. Therefore, only two cases in our cohort underwent the NGS panel. Targeted NGS was performed using the John Hopkins Genomics Molecular Diagnostics Laboratory clinical solid tumor panel. Capture-based NGS was performed using an assay that targets coding exons of 432 cancer-related genes (Supplementary Table S1). Libraries were prepared using the SureSelect^XT^ Target Enrichment System (Agilent Technologies, Santa Clara, CA, USA). Captured libraries were sequenced as paired-end reads on an Illumina HiSeq 2500 instrument (Illumina Biotechnology, San Diego, CA, USA). Sequence reads were mapped to the reference human genome build CRCh37 (hg19) using the Burrows–Wheeler aligner (BWA) v0.7.10. Variant filtering and calling were performed using the MDLVC v.10 pipeline. The capture-based NGS was performed using an assay that targets DNA segments at regular intervals along each chromosome to enable genome-wide copy number and zygosity analysis, including chromosomes 1p, 19q, 7, and 10 status.

## 3. Case Presentation

In this retrospective case series of nine patients, seven male and two female patients had a surgical resection or biopsy of IVGBM at our institution. The median age at diagnosis was 64.3 years, ranging from 36 to 85 years. A summary of patient characteristics is presented in Table 1. Seven patients presented with signs and/or symptoms of hydrocephalus. Confusion was the most reported symptom (n = 6, 67%), with additional presenting symptoms including memory deficits (5, 56%), and headache (4, 44%). One patient presented with a seizure and one patient presented with a transient ischemic attack. The average Karnofsky Performance Score (KPS) at presentation was 80 (SD: ±4).

**Figure 1:**
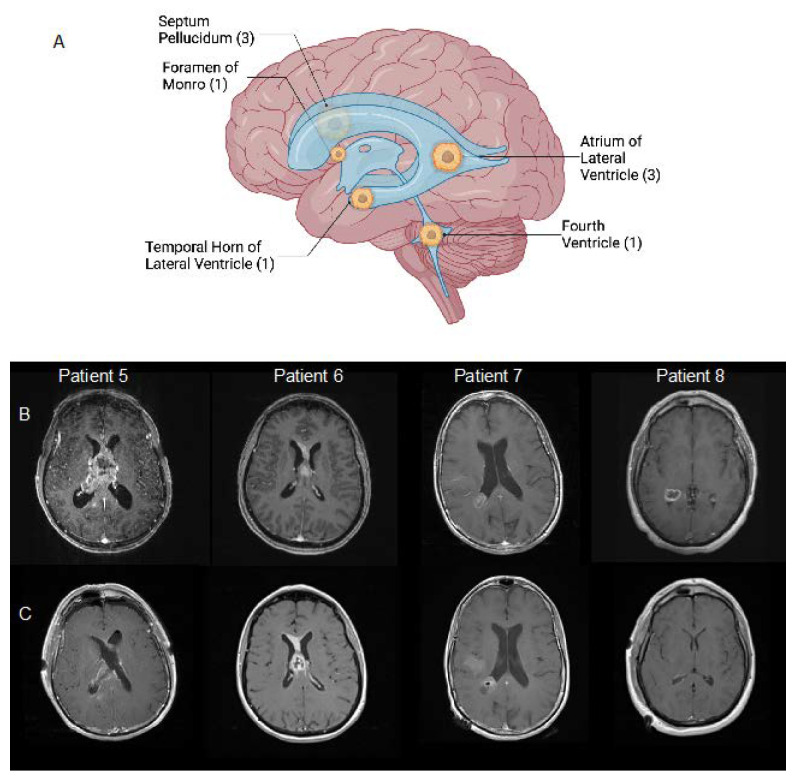
Locations of IVGBM in our cohort created with Biorender.com (**A**). T1 post-contrast magnetic resonance images showing (**B**) IVGBM prior to resection and (**C**) postoperatively in 4 patients with available radiographic images. Patients 5 and 7 received subtotal resection. Patient 6 received biopsy. Patient 8 received gross total resection.

Radiologic workup for our patients included both MRI and CT. The most common tumor locations were the atrium of the right lateral ventricle (3, 33%) and the septum pellucidum (3, 33%). Five patients had hydrocephalus radiographically, including three that required intraoperative external ventricular drains (EVD) and one that required a postoperative ventriculoperitoneal (VP) shunt. Obstructive hydrocephalus was managed in one patient with partial resection of the IVGBM. The ventricular locations of tumors, as well as preoperative and postoperative findings for patients with available MRI images, are presented in Figure 1.

One patient (patient 6) had a biopsy without subsequent tumor resection, six had subtotal resections, and two had gross total resections. The most common surgical approach was transcortical. For the eight patients who received tumor resection, entry into the ventricle either with the endoscope or the microscope via transcortical or transcallosal approach was the first step of resection. The IVGBMs were visualized upon entering the ventricles. For patient 1, a small craniectomy was performed at the base of the temporal fossa. Self-retaining retractors were used to open the superotemporal sulcus, allowing entry into the temporal horn of the right lateral ventricle where the mass was immediately visualized. A transcallosal approach was utilized for patient 2 and the tumor was immediately identified within the central portion of the lateral ventricles. The tumor was internally decompressed and then dissected free from the ventricular walls. Gross total resection was achieved and an EVD was placed intraoperatively. In the perioperative period, patient 2 gradually deteriorated. The EVD was opened up and did not show evidence of blood. For patient 3, a cortical incision was made at the junction between the cerebellar hemisphere and the vermis. Once the obex was identified, the tumor could be seen coming out of the floor of the fourth ventricle. The tumor was partially resected. For patient 4, a frontal transcortical approach was used to enter the right lateral ventricle. The microscope and intraoperative navigation were used to identify the mass within the septum pellucidum. Bipolar suction and tumor forceps were used to achieve subtotal resection of the tumor. An EVD was placed in the right lateral ventricle. For patient 5, a small frontal corticectomy was performed to enter the frontal horn of the ventricle. The tumor was identified medially in the septum pellucidum and the ultrasonic aspirator was used to achieve subtotal resection. An EVD was placed in the contralateral occipital horn intraoperatively. Postoperative MRI revealed resolution of the hydrocephalus and the EVD was removed on postop day 5. Patient 6 received a stereotactic needle biopsy. Three months following the biopsy, the patient presented with deterioration in consciousness secondary to obstructive hydrocephalus from the tumor. She received bilateral Strata^TM^ VP shunts set at 1.5. Patient 7 received a stereotactic needle biopsy of the tumor in the atrium of the right lateral ventricle and subtotal resection of a second lesion identified in the parenchyma posterior to the lateral ventricle. For patient 8, the right lateral ventricle was accessed via the middle temporal sulcus. Normal white matter overlying the tumor capsule was gently bipolar cauterized and the highly vascular tumor was immediately apparent. The tumor was debulked until normal white matter was visualized in all directions. A corticectomy was used to access the ventricular system of patient 9. The tumor in the atrium of the right lateral ventricle was partially resected. Hemostasis was important in all cases in order to prevent intraventricular hemorrhage. No intraoperative complications were noted.

The average hospital length of stay in our cohort was 12.1 days (SD: ±10.5 days). Five patients were discharged home, two were discharged to a rehabilitation center, and one was discharged to hospice. One patient (patient 1) died on postoperative day 36 after receiving treatment for atelectasis, pneumonia, sepsis, pulmonary embolism, renal insufficiency, and severe hypotension.

**Figure 2:**
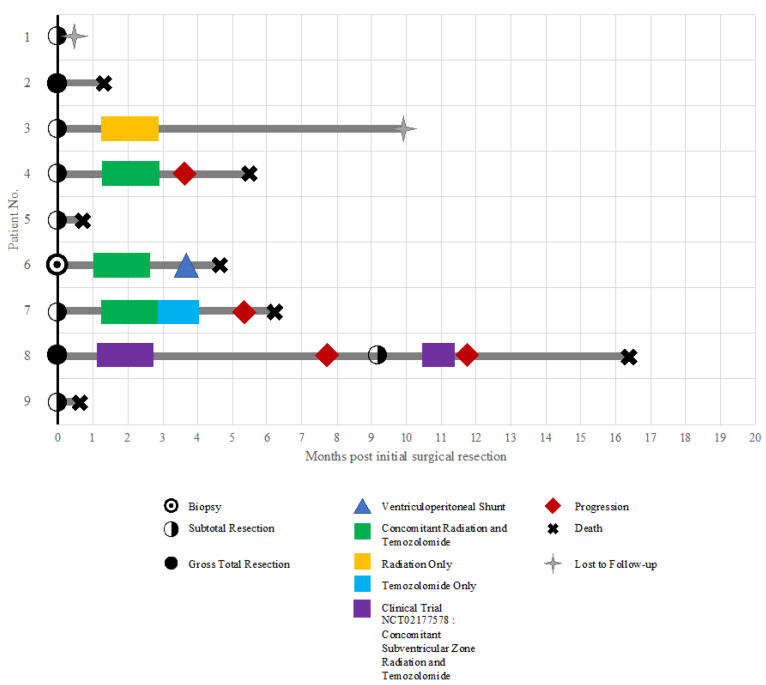
Swimmer’s plot showing timing of treatment and clinical outcomes in our cohort of 9 patients with IVGBM.

Follow-up data were only available for seven patients. Following surgical resection, five patients received adjuvant therapy. The average time between initial surgical resection and the start of adjuvant therapy was 1.21 months (range: 1.00–1.43 months). Three patients (patients 4, 6, and 7) received standard-of-care radiation therapy to a total dose of 60 cGy with concomitant temozolomide. However, no patients received an additional six cycles of temozolomide to complete the Stupp protocol [23]. Following 6 weeks of chemoradiation, patient 7 received two cycles of temozolomide, but discontinued treatment due to tumor recurrence as well as seeding in the leptomeninges and the ependymal lining of the lateral and third ventricles. This patient had rapid progression and died six weeks later. Two patients did not receive maintenance temozolomide after 6 weeks of concomitant radiation and chemotherapy. This was due to progressively worsening left-sided hemiplegia in one patient (patient 4), whereas the other patient (patient 6) had bilateral deep-vein thromboses, pulmonary emboli, and thrombocytopenia secondary to chemotherapy. One patient (patient 8) received concomitant subventricular zone (SVZ) irradiation and temozolomide for two months under a clinical trial (NCT02177578) at our institution. Patient 3 received 32 days of radiotherapy without concomitant chemotherapy. Three patients did not receive adjuvant therapy due to rapid disease progression and compromised functional status. Additional details of the timing of clinical management and disease progression for our cohort are depicted in Figure 2. There was no significant difference in the KPS at diagnosis between patients who received adjuvant therapy and patients who did not.

**Figure 3:**
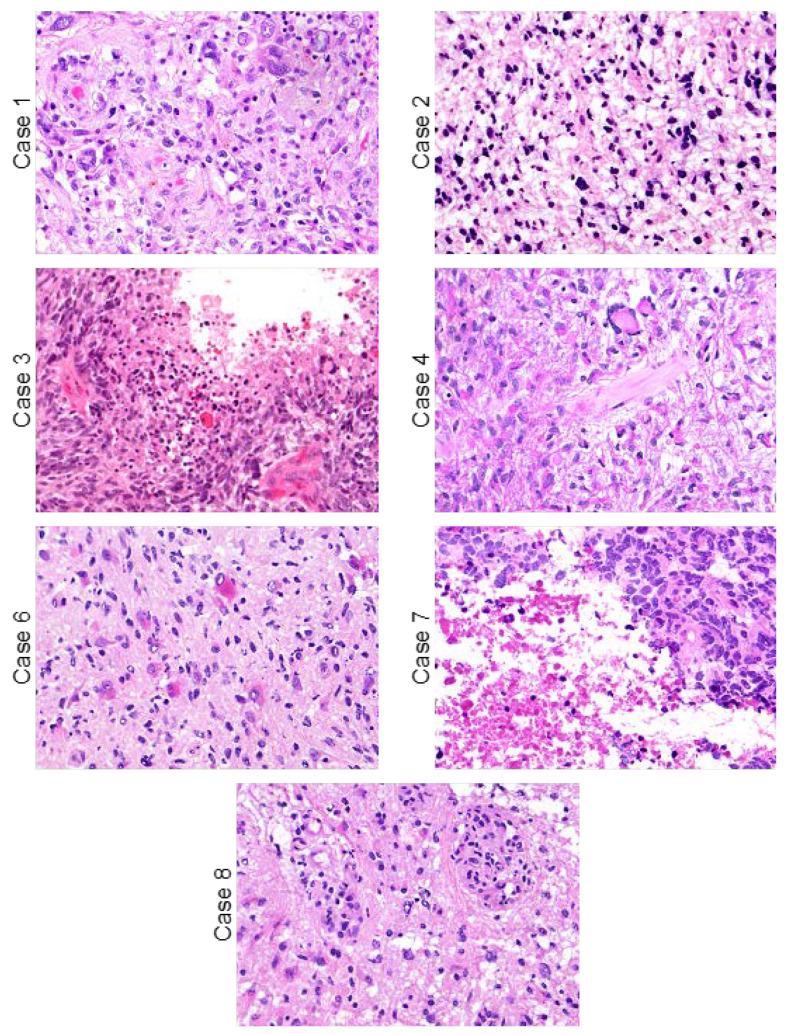
H&E images of tumors with available FFPE blocks captured via microscope camera. Microscope magnification was 40× for all images.

Our search criteria of the surgical pathology records, which included “glioblastoma”, “intraventricular”, and “neurosurgical intervention” identified nine cases. The diagnosis of GBM was histologically confirmed in all cases based on the presence of sheets of atypical glial cells with an increased mitotic rate (>5 mitotic figures per 10 high-power fields) (Figure 3). Eight cases additionally demonstrated a combination of necrosis and/or microvascular proliferation and received the diagnosis of GBM, WHO Grade IV according to the WHO 2016 tumor classification criteria. Patient 2’s tumor was classified as glioblastoma in the initial surgical pathology report and in clinical records. Of note, when re-reviewed by our neuropathologist, this tumor was reclassified as grade III according to the WHO 2016 tumor classification, given the lack of necrosis and microvascular proliferation. Immunohistochemistry for IDH1 R132H mutant protein was performed in four cases and was negative in all four (patients 6, 7, 8, and 9). All available histopathological data are presented in Table 2. NGS and MGMT promoter methylation testing were performed on resected tumor tissue for patients 6 and 8 (Table 3). IDH1 mutations were not detected by NGS in both cases, confirming the diagnoses of GBM, IDH-wildtype, WHO Grade IV. Genetic alterations common in GBM, IDH-wildtype, were seen in these two cases, including EGFR gene amplification, TERT promoter mutation, PTEN mutation, trisomy of chromosome 7, and monosomy of chromosome 10. MGMT promoter methylation was negative in both tumors. Additional mutations of unknown significance in these two tumors are reported in Supplementary Table S2.

Median survival among our cohort was 4.7 months (IQR: 0.9–5.8 months). The longest survival was 16.3 months. This patient (patient 8) had a 2.4 cm × 2 cm × 3.5 cm tumor in the atrium of the right lateral ventricle. The patient was enrolled in a clinical trial (NCT02177578), and after undergoing surgery for gross total resection, the patient received concomitant subventricular zone (SVZ) irradiation and temozolomide for two months and was disease-free for 8 months. The tumor recurred locally and a repeat subtotal resection was performed. This patient received one month of concomitant SVZ irradiation and temozolomide under the same clinical trial following the repeat resection. No other patients in this cohort received SVZ radiation. Tumor progression and increased surrounding edema were observed on MRI two months following repeat resection. The patient died four months after discontinuing this therapy due to local tumor progression.

## 4. Discussion

IVGBMs are rare and there is limited guidance on their management. Previous reports have designated a tumor as intraventricular if the tumor is attached to the ventricular walls, is located primarily within the ventricular system, and causes local ventricular expansion as it grows [16,24]. While the rarity of these tumors precludes a precise understanding of their pathogenesis, the septum pellucidum, fornix, and pluripotent stem cells within the subependymal zone have been identified as potential origin sites of glial proliferation into the ventricles [25,26]. Alternatively, the spread of cerebral GBM through the cerebrospinal fluid has been described in 10–20% of cases [27,28,29].

Management of IVGBMs is particularly challenging because they are difficult to access surgically and can present with obstructive hydrocephalus, necessitating prompt intervention [3,4,5,6,7,8,9,10,11,12,13,14,15,16,17]. In prior reports of adults with IVGBM, the average age at presentation was approximately 48 years, whereas the average age at presentation in our cohort was approximately 64 years [3,4,5,6,7,8,9,10,11,12,13,14,15,16,17]. The average age at presentation in adult patients with parenchymal GBM is 64 years [2]. IVGBMs have been documented throughout the ventricular system, with the majority located in the body of the lateral ventricle, the trigone (atrium) of the lateral ventricle, and the anterior third ventricle, respectively [3,4,5,6,7,8,9,10,11,12,13,14,15,16,17]. The majority of IVGBMs in our cohort were located within the trigone of the lateral ventricle and the septum pellucidum. Reports of IVGBM arising from the septum pellucidum are notably rare in the literature, and resection is typically carried out via a transcortical or transcallosal approach [10,16]. Remarkably, gross total resection of IVGBM arising from the septum pellucidum has been rarely documented. In some of the documented cases, the transcallosal approach was the approach of choice for resection [10,16]. In the three patients with IVGBM arising from the septum pellucidum in our cohort, the transcortical approach was used to achieve subtotal resection in two patients and the transcallosal approach was used to achieve gross total resection in one patient. Intraoperative hemostasis was critical for preventing intraventricular hemorrhage for all resections. We also observed IVGBM in the foramen of the Monro and the fourth ventricle. Descriptions of third ventricular and foraminal IVGBM are limited to case reports, and IVGBM is extremely rare in the fourth ventricle [14,30,31].

While gross total resection has been shown to prolong overall survival compared to subtotal resection in patients with parenchymal gliomas, the impact of the extent of resection on overall survival in IVGBM is unclear due to the modest number of reported cases [32]. In the present series, gross total resection was achieved in two patients. One patient failed extubation after gross total resection of a GBM located in the septum pellucidum, whereas the other patient who underwent gross total resection survived for 16 months. The median survival time of our cohort was approximately 5 months, which is substantially shorter than the median survival times reported for parenchymal GBM [1,2]. Only one patient in our cohort survived longer than 15 months, which more closely approximates the median survival time in patients with GBM in other cerebral locations after completing standard-of-care therapy [33]. This was the patient enrolled in a clinical trial involving radiation of the SVZ.

Although we do not have enough power to make statistically significant associations, we see that the majority of patients in our cohort with IVGBM have complicated clinical courses with the inability to complete standard-of-care adjuvant therapies. Radiation of the SVZ may be a promising alternative for patients with IVGBM [34,35]. The SVZ, which lies between the corpus callosum, lateral ventricle, and striatum houses the largest population of neural stem cells in the brain and has been implicated in GBM tumorigenesis [36,37]. Notably, the extension of the tumor into the SVZ is associated with lower overall survival and progression-free survival [38,39,40]. Additionally, studies have shown that tumors contacting the SVZ are more likely to recur aggressively, be of larger size, cross the midline, be multifocal, and may have increased proliferation, as evidenced by increased methionine positron emission tomography, than tumors that do not contact the ventricle [41]. SVZ contact has also been associated with the lack of MGMT promoter methylation, a poor prognostic factor in diffuse gliomas [38]. In patients with parenchymal GBM, a mean radiation dose of 40 Gy or greater to the ipsilateral SVZ was associated with improved progression-free survival and overall survival [41,42]. In 2021, Iacoangeli et al. reported a case of one patient with an IVGBM who was treated with a neuroendoscopic surgical approach and received intrathecal chemotherapy through an intraventricular catheter [43]. The role of SVZ radiation and intrathecal chemotherapy in the management of IVGBM warrants further investigation.

Other reports of survival in patients with intraventricular adult diffuse gliomas are varied and confounded by differences in molecular markers such as IDH-mutant status, especially given the evolving molecular classification parameters of diffuse gliomas. It is well established that IDH-wildtype gliomas are more fast-growing and associated with a worse prognosis than IDH-mutant gliomas [44]. In our cohort, all four patients with available immunohistochemistry results had IDH-wildtype tumors.

Alterations captured by NGS in two patients were consistent with molecular alterations commonly observed in GBM, including EGFR amplification and mutations in PTEN and NF1 [45,46,47,48]. EGFR amplification has been observed in 57% of GBM and is associated with enhanced tumor cell angiogenesis [49]. Indeed, EGFR mutation is the target of many investigational therapies. PTEN and NFI mutations are also well described in GBM [48]. Zhu et al. demonstrated that NFI loss may cooperate with PTEN and p53 inactivation in the development of malignant glioma [49]. Notable alterations in patient 6 also included BCOR and TSC2. The BCOR gene has been shown to be altered in a subset of pediatric tumors with embryonal features, as well as in pediatric gliomas [50,51]. Both NFI and TSC2 are tumor suppressor genes associated with autosomal dominant tumor predisposition syndromes that increase the risk for CNS neoplasms in addition to a constellation of other syndromic features [52,53]. In patient 8, notable alterations were BRCA2, RB1, and SETD2. BRCA2 is a tumor suppressor gene that is commonly implicated in breast and ovarian cancers but has also been associated with poor prognosis in gliomas [54]. Conversely, the alteration of RB1, which is a tumor suppressor gene associated with retinoblastoma and osteogenic sarcoma, may be associated with a better prognosis in GBM [55]. Finally, the alteration of SETD2, which encodes a histone methyltransferase, has been associated with resistance to chemotherapy in GBM [56].

Other studies describing the molecular landscape of IVGBM are limited. Takigawa et al. detected TP53 and NFKBIA mutations via sequencing polymerase chain reaction analysis of DNA extracted from an IDH-wildtype mucin-producing IVGBM [57]. TP53 mutations are frequently associated with diffuse astrocytoma, which typically have a more indolent clinical course than IDH-wildtype GBM [56]. The patient documented by Takigawa et al. remained clinically stable for five years following resection.

Due to differences in the location and extent of resection of these tumors, the limited number of patients with available sequencing data, and incomplete survival data, we are unable to report an association between the genetic characteristics of IVGBM in our cohort and overall survival. In addition, the lack of IDH mutation status for all our patients is a limitation given the current WHO classification. We recognize that additional immunohistochemistry and NGS data could change the characterization of the tumors included in our cohort. Nevertheless, we present valuable next-generation sequencing data from two IVGBMs, which are extremely rare and have not been molecularly well-characterized. Additional studies are needed to better understand the genetic landscape of IVGBM compared to parenchymal GBM and may further elucidate the unique pathophysiology of these rare tumors.

## 5. Conclusions

Despite genetic similarities to parenchymal GBM, management of our cohort of IVGBM was complicated by difficult surgical approaches, complex clinical courses that may have precluded completion of standard-of-care therapies, and higher postoperative morbidity. These considerations may help guide patient counseling and management when IVGBM is suspected. While we present valuable genetic data for two IVGBM, which are rare, the genetic results of this small cohort should be confirmed in larger studies. Further, multi-institutional registries and meta-analyses are needed to expand on our findings and better elucidate the unique pathophysiology of these rare intraventricular brain tumors.

## Figures and Tables

**Table 1:** Cohort characteristics and outcomes.

Patient	Age, y	Sex	Presenting Symptoms	Location	Surgical Approach	Extent of Resection	Shunt/Drain	Postoperative Complications	Postop LOS (Days)	Adjuvant Therapy	Overall Survival [1]
1	57	M	Seizures, headaches	Temporal horn of right lateral ventricle	Right temporal transcortical	STR	None	Unknown	3	Unknown	Lost to follow-up
2	65	M	Confusion, memory deficit	Septum pellucidum	Right anterior transcallosal	GTR	EVD	Prolonged intubation, seizure, pulmonary embolism, sepsis, renal insufficiency, atelectasis	36	None	36 days
3	37	M	Headache, ophthalmoplegia, facial droop, ataxia	Fourth ventricle	Suboccipital transvermian	STR	None	Temporary swallowing deficit with PEG, hemiplegia, new cognitive deficit	18	RT only (5840 cGy)	Lost to follow-up
4	71	M	Confusion, memory deficit	Septum pellucidum	Right frontal transcortical	STR	EVD	Pulmonary emboli, NSTEMI	17	RT (6000 cGy)/TMZ	5 months
5	77	M	Confusion, memory deficit	Septum pellucidum	Right frontal transcortical	STR	EVD	Prolonged coma	12	None	19 days
6	61	F	Headache, confusion, memory deficit, sleepiness	Foramen of Monro	Stereotactic needle biopsy	Biopsy	VP shunt	Pulmonary embolism; intracranial hemorrhage	4	RT (6000 cGy)/TMZ	4 months
7	68	F	Transient ischemic attack	Atrium of right lateral ventricle	Right parietal transcortical	STR	None	Hearing loss	2	RT (6000 cGy)/TMZ + TMZ x2 cycles	5 months
8	56	M	Confusion	Atrium of right lateral ventricle	Right temporal transcortical	GTR	None	None	2	:SVZ RT (6000 cGy)/TMZ [2]	16 months
9	69	M	Headaches, nausea/vomiting, confusion, memory deficit	Atrium of right lateral ventricle	Right anterior transcallosal	STR	None	New sensory deficit	15	None	18 days

EVD, external ventricular drain; F, female; GTR, gross total resection; LOS, length of stay; M, male; NSTEMI, non-ST-elevation myocardial infarction; PEG, percutaneous endoscopic gastrostomy; STR, subtotal resection; SVZ, subventricular zone; RT, radiotherapy; TMZ, temozolomide; VP, ventriculoperitoneal. [1] Survival time following date of surgery for pathological confirmation of GBM. [2] Clinical trial NCT02177578.

**Table 2:** Histopathological Characteristics.

Patient	Year of Surgery	Initial Diagnosis	WHO 2016 Grade	MVP	Necrosis	Mitotic Rate	IDH-1 R132H IHC	ATRX IHC	P53 IHC	1p/19q
1	1995	Glioblastoma	IV	+	+	Increased	NP	NP	NP	NP
2	1998	Glioblastoma [1]	III	−	−	Increased	NP	NP	NP	NP
3	2002	Glioblastoma	IV	+	+	Increased	NP	NP	NP	NP
4	2009	Glioblastoma	IV	+	+	Increased	NP	NP	NP	NP
5	2006	Glioblastoma	IV	+	+	Increased	NP	NP	NP	NP
6	2019	Glioblastoma	IV	+	+	Increased	Negative	Retained	Wildtype	Intact on NGS
7	2017	Glioblastoma	IV	−	+	Increased	Negative	Retained	Wildtype	NP
8	2021	Glioblastoma	IV	+	−	Increased	Negative	Retained	Wildtype	Intact on NGS
9	2014	Glioblastoma	IV	−	+	Increased	Negative	NP	NP	NP

IHC, immunohistochemistry; MVP, microvascular proliferation; NP, not performed. [1] This tumor was originally characterized as glioblastoma at the time of pathological diagnosis and in the clinical records. Notably, on re-review by our senior neuropathologist this tumor was given the grade III designation due to the absence of necrosis and microvascular proliferation, according to the WHO Classification of Central Nervous System Tumors.

**Table 3:** Oncogenic variants identified in two cases with available NGS data.

Variant	RefSeq Transcript	Chromosome	Genomic Position	Reference Allele	Alternate Allele	Function	Sequencing Depth	Mutant Allele Frequency
**Patient 6**								
PTEN p.N292fs	NM_000314	chr10	g.89,720,720	GA	G	frameshift	262	62%
NF1 p.Y1659fs	NM_000267	chr17	g.29,653,035	ATATC	A	frameshift	1169	33%
EGFR focal amplification	NM_005228	chr7	whole gene amplification					
Chromozome (7+/10−)								
**Patient 8**								
TERT c.-146C>T	NM_198253	chr5	g.1,295,250	G	A	upstream	620	35%
PTEN p.C124Y	NM_000314.7	chr10	g.89,692,887	G	A	missense	220	46%
Chromosome (7+/10−)								

## Data Availability

The data presented in this study are available on request from the corresponding author. The data are not publicly available in order to maintain patient privacy.

## Supplementary Materials

The supporting information can be downloaded at: https://www.mdpi.com/article/10.3390/ijms241713285/s1, Table S1: Complete Genetic Mutation Profile of Two Patients; Table S2: Genes covered by the Johns Hopkins Genomics Molecular Diagnostics Laboratory clinical solid tumor panel.
