# Supplementary material for: Management and Molecular Characterization of Intraventricular Glioblastoma: A Single-Institution Case Series
Source: Int J Mol Sci. 2023 Aug 27;24(17):13285. doi: 10.3390/ijms241713285 (PMC10488126; doi:10.3390/ijms241713285)
Supplement: Supplementary file 1 [file ijms-24-13285-s001.zip › Supplementary Table S1 8.26.pdf]

Supplemental Table S1. Genes covered by the Johns Hopkins Genomics Molecular Diagnostics Laboratory clinical solid tumor panel

ABL1	BTK	CYLD	FGF23	HIST1H2BJ	LRRK2	NPM1	RAD21	STAT4
ABL2	BTLA	DAXX	FGF3	HIST1H2BK	MAF	NRAS	RAD50	STAT5A
ACTB	CAD	DDR2	FGF4	HIST1H2BO	MAFB	NSD1	RAD51	STAT5B
ACVR1B	CALR	DDX3X	FGF6	HIST1H3B	MAGED1	NT5C2	RAF1	STAT6
AKT1	CARD11	DEK	FGFR1	HNF1A	MALT1	NTRK1	RARA	STK11
AKT2	CBFB	DICER1	FGFR2	HRAS	MAP2K1	NTRK2	RASGEF1A	SUFU
AKT3	CBL	DNM2	FGFR3	HSP90AA1	MAP2K2	NTRK3	RB1	SUZ12
ALK	CBLB	DNMT3A	FGFR4	ICK	MAP2K4	NUP93	RECQL4	SYK
APC	CCND1	DOT1L	FH	ID3	MAP3K1	NUP98	RELN	SYNE1
APH1A	CCND2	DTX1	FHIT	IDH1	MAP3K14	P2RY8	RET	TAF1
AR	CCND3	DUSP2	FLCN	IDH2	MAP3K6	PAG1	RHOA	TBL1XR1
ARAF	CCNE1	DUSP9	FLT1	IGF1R	MAP3K7	PAK3	RHOH	TCF3
ARFRP1	CCT6B	EBF1	FLT3	IGF2	MAPK1	PALB2	RICTOR	TCL1A
ARHGAP26	CD22	ECT2L	FLT4	IKBKE	MCL1	PASK	RNF43	TET2
ARID1A	CD274	EED	FLYWCH1	IKZF1	MDM2	PAX5	ROS1	TGFBR2
ARID1B	CD36	EGFR	FOXL2	IKZF2	MDM4	PBRM1	RPTOR	TLL2
ARID2	CD58	ELP2	FOXO1	IKZF3	MED12	PC	RUNX1	TMEM30A
ASMTL	CD70	EML4	FOXO3	IL7R	MEF2B	PCBP1	RUNX1T1	TNFAIP3
ASXL1	CD79A	EP300	FOXP1	INHBA	MEF2C	PCLO	S1PR2	TNFRSF11A
ATM	CD79B	EPHA3	FRS2	INPP4B	MEN1	PDCD11	SDHA	TNFRSF14
ATR	CDC73	EPHA5	FUBP1	INPP5D	MET	PDCD1LG2	SDHB	TNFRSF17
ATRX	CDH1	EPHA7	GADD45B	IRF1	MIB1	PDGFRA	SDHC	TOP1
AURKA	CDK12	EPHB1	GATA1	IRF4	MITF	PDGFRB	SDHD	TP53
AURKB	CDK4	ERBB2	GATA2	IRF8	MKI67	PDK1	SERP2	TP63
AXIN1	CDK6	ERBB3	GATA3	IRS2	MLH1	PHF6	SETBP1	TRAF2
AXL	CDK8	ERBB4	GID4	JAK1	MPL	PIGA	SETD2	TRAF3
B2M	CDKN1B	ERG	GNA11	JAK2	MRE11A	PIK3C2B	SF3B1	TRAF5
BAP1	CDKN2A	ESR1	GNA12	JAK3	MSH2	PIK3CA	SGK1	TRAF7
BARD1	CDKN2B	ETS1	GNA13	JARID2	MSH3	PIK3CB	SMAD2	TSC1
BCL10	CDKN2C	ETV6	GNAQ	JUN	MSH6	PIK3CG	SMAD4	TSC2
BCL11B	CEBPA	EXOSC6	GNAS	KAT6A	MTOR	PIK3R1	SMARCA1	TSHR
BCL2	CHD2	EZH2	GPR124	KDM2B	MUTYH	PIK3R2	SMARCA4	TUSC3
BCL2L1	CHEK1	FAF1	GRIN2A	KDM4C	MYC	PIM1	SMARCB1	TYK2
BCL2L2	CHEK2	FAM46C	GSK3B	KDM5A	MYCL1	PLCG2	SMC1A	U2AF1
BCL6	CIC	FANCA	GTSE1	KDM5C	MYCN	PML	SMC3	U2AF2
BCL7A	CIITA	FANCC	H3F3A	KDM6A	MYD88	PMS2	SMO	VHL
BCOR	CKS1B	FANCD2	HDAC1	KDR	MYO18A	POT1	SOCS1	WDR90
BCORL1	CPS1	FANCE	HDAC4	KEAP1	NCOR2	PPP2R1A	SOCS2	WHSC1
BCR	CREBBP	FANCF	HDAC7	KIT	NCSTN	PRDM1	SOCS3	WISP3
BIRC3	CRKL	FANCG	HGF	KLF4	NF1	PRKAR1A	SOX10	WT1
BLM	CRLF2	FANCL	HIST1H1C	KLHL6	NF2	PRKDC	SOX2	XBP1
BRAF	CSF1R	FAS	HIST1H1D	KMT2A	NFE2L2	PRSS8	SOX9	XPO1
BRCA1	CSF3R	FBXO11	HIST1H1E	KMT2B	NFKBIA	PTCH1	SPEN	YY1AP1
BRCA2	CTCF	FBXO31	HIST1H2AC	KMT2D	NKX2-1	PTEN	SPOP	ZMYM3
BRD4	CTNNA1	FBXW7	HIST1H2AG	KRAS	NLRP1	PTPN11	SRC	ZNF217
BRIP1	CTNNB1	FGF10	HIST1H2AL	LEF1	NOD1	PTPN2	SRSF2	ZNF24
BRSK1	CUX1	FGF14	HIST1H2AM	LMO1	NOTCH1	PTPN6	STAG2	ZNF703
BTG2	CXCR4	FGF19	HIST1H2BC	LRP1B	NOTCH2	PTPRO	STAT3	ZRSR2
