# Supplementary material for: Management and Molecular Characterization of Intraventricular Glioblastoma: A Single-Institution Case Series
Source: Int J Mol Sci. 2023 Aug 27;24(17):13285. doi: 10.3390/ijms241713285 (PMC10488126; doi:10.3390/ijms241713285)
Supplement: Supplementary file 1 [file ijms-24-13285-s001.zip › Supplementary Table S2 8.26.pdf]

Supplementary Table S2. Complete Genetic Mutation Profile of Two Patients				
**Patient 6**				
**Gene**	**Amino Acid Change**	**Variant Allele Frequency**	**Copy Number Variation**	**Mutation Status**
**ATM**	p.F1463C	54.86		Benign/Likely Benign
**NF1**	p.Y1659fs	32.57		Pathogenic/Likely Pathogenic
**PMS2**	p.A572V	61.42		VUS
**PTEN**	p.N292fs	62.4		Pathogenic/Likely Pathogenic
**TSC2**	p.P768S	54.8		VUS
**BCOR**	p.E1093K	43.9		VUS
**CARD11**	p.V429I	24.48		VUS
**FBXO11**	p.E234K	48.18		VUS
**KDR**	p.R1022*	39.5		VUS
**EGFR**			Amplified	Pathogenic/Likely Pathogenic
**Patient 8**				
**Gene**	**Amino Acid Change**	**Variant Allele Frequency**	**Copy Number Variation**	**Mutation Status**
**BRCA2**	p.D2237V	73.59		VUS
**PTEN**	p.C124Y	45.89		Pathogenic/Likely Pathogenic
**RB1**	p.L662R	43.51		VUS
**TERT**	c.-146C>T	35.02		Pathogenic/Likely Pathogenic
**EPHB1**	p.S774L	43.23		VUS
**FANCE**	p.Q182fs	29.7		VUS
**KDM5A**	p.I1226V	52.77		VUS
**PCLO**	p.R3540W	22.31		VUS
**SETD2**	p.A2451V	47.14		VUS
**WDR90**	p.R784Q	45.61		VUS
***Stop Codon**				
